# A Novel Risk Score Facilitates Femoral Artery Access in Transcatheter Aortic Valve Implantation: Passage-Puncture Score

**DOI:** 10.1016/j.shj.2024.100331

**Published:** 2024-06-25

**Authors:** Mi Chen, Jonathan Michel, Barbara E. Stähli, Christian Templin, Philipp Jakob, Thomas S. Gilhofer, Felix C. Tanner, Albert Markus Kasel

**Affiliations:** Department of Cardiology, University Heart Center, University Hospital of Zurich, University of Zurich, Zurich, Switzerland

**Keywords:** Transcatheter aortic valve implantation, Transfemoral approach, Vascular closure device, Vascular complication

## Abstract

**Background:**

Vascular complications remain high in transfemoral transcatheter aortic valve implantation (TAVI). Careful evaluation of the femoral arteries is important to select the optimal access site.

**Objectives:**

This study sought to describe a novel risk score (the passage-puncture score) for transfemoral access using a single suture-based closure system.

**Methods:**

The passage-puncture score consists of the evaluation of 1) passage feasibility of the ilio-femoral arteries (passage score) and 2) puncture site feasibility (puncture score) based on pre-TAVI computed tomography. All patients underwent fluoroscopy-guided arterial puncture and closure with a suture-based closure system. The primary endpoint was the rate of vascular complications in discharge, including minor and major vascular complications according to the definitions of the Third Valve Academic Research Consortium.

**Results:**

From September 2020 to June 2021, transfemoral TAVI was performed in 98 of 99 patients. Passage score (right) was significantly higher in patients treated by left compared to those treated by right femoral access (3 vs. 1; *p* <0.001). Puncture score was significantly different between patients undergoing mid-femoral as compared to nonmid-femoral puncture (0 vs. 3, *p* <0.001). Minor vascular complications occurred in six (6%) patients.

**Conclusions:**

The passage-puncture score is effective in defining the optimal access site for transfemoral TAVI. The systematic evaluation has the potential to further reduce access-site complications.

## Highlights

• It is advised to conduct a systematic evaluation of femoral access using computed tomography.
• The Passage-Puncture Score is an effective tool for determining optimal access points.
• Employing a single ProGlide technique can achieve satisfactory outcomes.

## Graphical abstract

**Figure:**
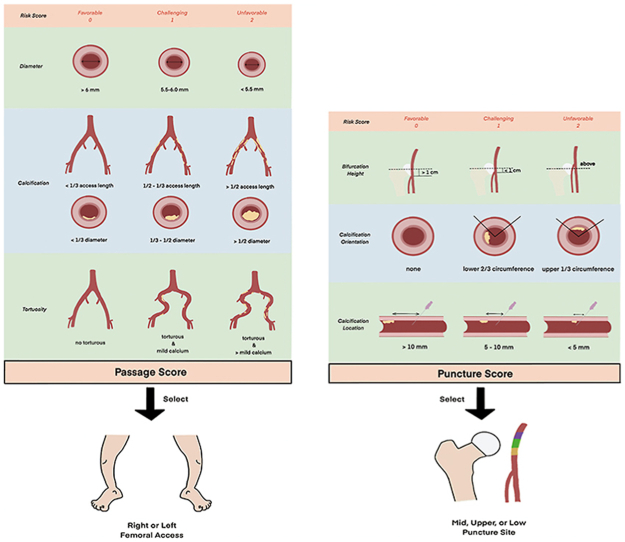

## Introduction

Vascular complications remain of major concern in transfemoral transcatheter aortic valve implantation (TAVI), with an estimated prevalence of about 4% to 10%.^1^ Patient characteristics, femoral artery dimensions, vessel calcifications, and tortuosity, along with sheath size and operator experience, determine vascular access site complications in TAVI.^2^^,^^3^ Reduced device profiles with consequently smaller sheaths and increasing operator experience with vascular closure devices (VCDs) are important factors that have contributed to a substantial decline in rates of vascular complications.^2^^,^^4^

A systematic vascular access site evaluation may further lower rates of vascular complications. Some studies showed that femoral artery depth, sheath-to-femoral-artery ratio, calcifications, and vessel tortuosity are associated with increased rates of vascular complications.^2^^,^^3^^,^^5^ However, a systematic evaluation for optimal access site selection in patients undergoing TAVI is lacking. We therefore developed an imaging-based, user-friendly score for systematic access site evaluation and puncture site selection in transfemoral TAVI.

## Methods

### Patient Population

This is a prospective, single-arm, single-center analysis of patients undergoing transfemoral TAVI using single-ProGlide at the University Hospital Zurich, Switzerland, between September 2020 and June 2021. All patients included in this study were enrolled in the prospective Zurich SwissTAVI Registry. As previously described, the SwissTAVI Registry is a national, multicenter cohort study initiated by the Swiss Working Group of Interventional Cardiology and the Swiss Society of Cardiac and Thoracic Vascular Surgery in 2011 (NCT01368250). In all patients, TAVI indication is confirmed by the institutional heart team and TAVI are performed according to current guidelines and recommendations. Central data monitoring and verification of data completeness is performed by an independent Clinical Trial Unit. All patients undergoing transfemoral TAVI were employed using single-ProGlide technique without anatomical selection. Exclusion criteria were transaortic, transcaval, or transsubclavian approaches. Demographic, baseline, and procedural characteristics, along with follow-up data, are systematically entered in a dedicated database. Follow-up was performed in-hospital, at 30 days, and yearly thereafter by means of standardized clinical visits or phone calls.

All patients underwent pre-TAVI computed tomography (CT) to evaluate the aortic annulus morphology and the feasibility of the transfemoral approach. In all patients, fluoroscopy-guided arterial puncture was performed, and a suture-based closure system was used for hemostasis.

### Passage-Puncture Score

All patients were evaluated with the TAVI-CT-based “passage-puncture score” to define^1^: the feasibility of transfemoral approach (“passage score”)^2^; the optimal vascular access side (right or left)^3^; the height of the puncture: upper, mid, or lower third of the femoral head as anatomical landmark (“puncture score”).

The “passage score” is used to evaluate the suitability of the access artery for device sheath insertion from the common femoral artery to the distal aorta. Four elements are assessed by TAVI-CT: 1) minimum lumen diameter; 2) calcification length; 3) maximal calcification thickness; and 4) vessel tortuosity (scored in the presence of concomitant vessel calcification and weighted by calcium quantity). A calcification score of 1 (based on length and/or thickness) is defined as “mild calcifications,” while a score more than 1 is defined as “moderate-to-severe calcifications.” Tortuosity is defined by either the presence of double iliac sign (whenever a part of the iliac artery is visualized more than once on any axial CT slice) or index of tortuosity.^6^ Each element can be scored with 0 points (favorable anatomy), 1 point (challenging anatomy), and 2 points (unfavorable anatomy). For the “passage score,” 0 to 8 points can be scored for each femoral artery.

**Figure 1:**
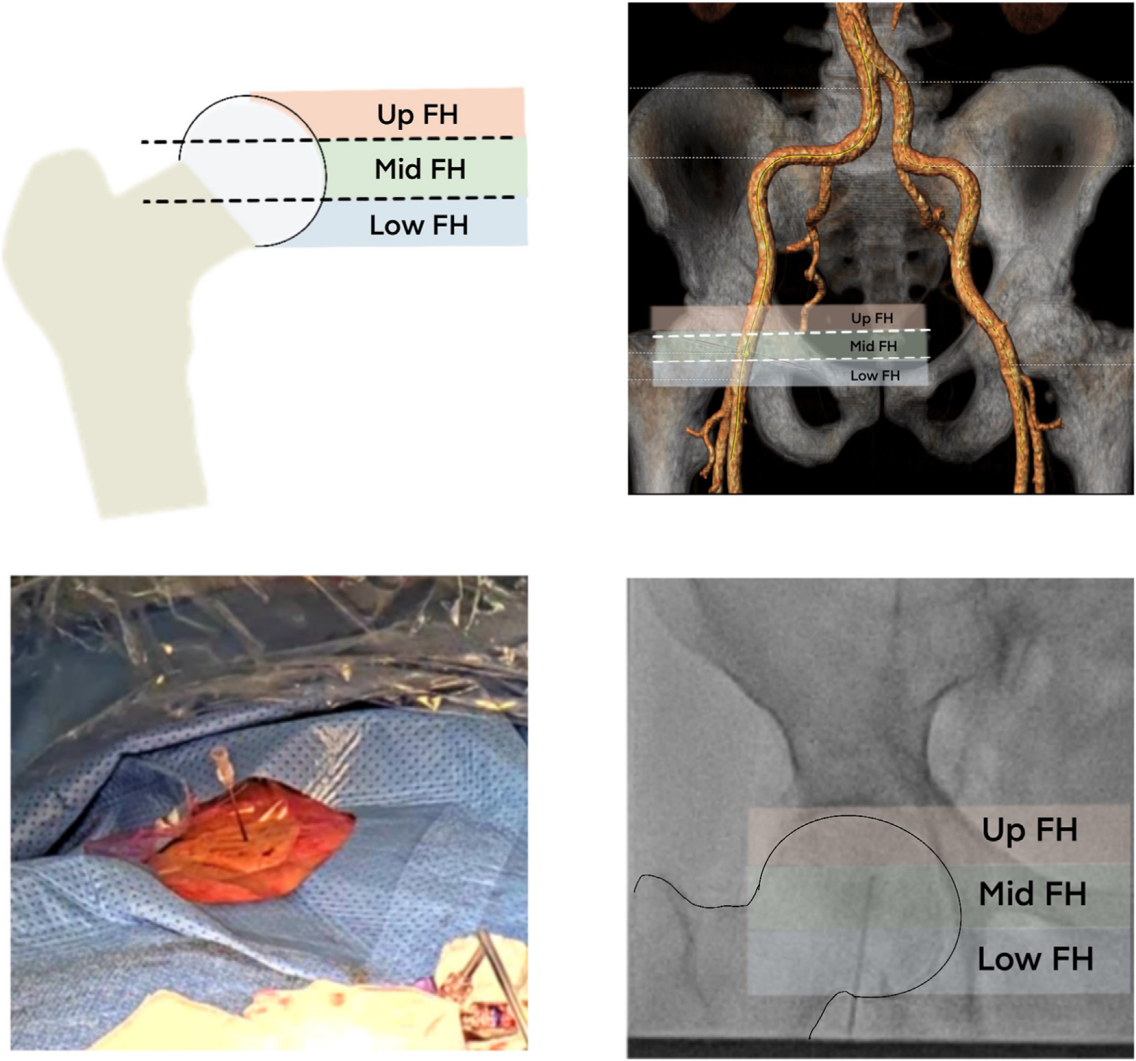
Integrated evaluation of the puncture site in computed tomography and fluoroscopy. The femoral head was divided into the upper, middle, and lower thirds. The paralleled three segments of femoral artery are the three puncture candidate sites. A 45-degree puncture needle marks the location under fluoroscopy and aims the predefined puncture site. Abbreviation: FH, femoral head.

The “puncture score” defines the optimized puncture height. Based on the TAVI-CT, the femoral artery is divided into 3 segments (upper, mid, and lower third, Figure 1). The bifurcation height of the femoral artery, calcification orientation, and calcification-free vessel length are assessed. Calcification-free length was defined as the distance between the predefined puncture site—the midpoint of the upper, mid, or lower artery segment—and the nearest calcium. Each element can be scored with 0 points (favorable anatomy), 1 point (challenging anatomy), and 2 points (unfavorable anatomy). For the “puncture score,” 0 to 6 points can be scored for each puncture site.

**Figure 2:**
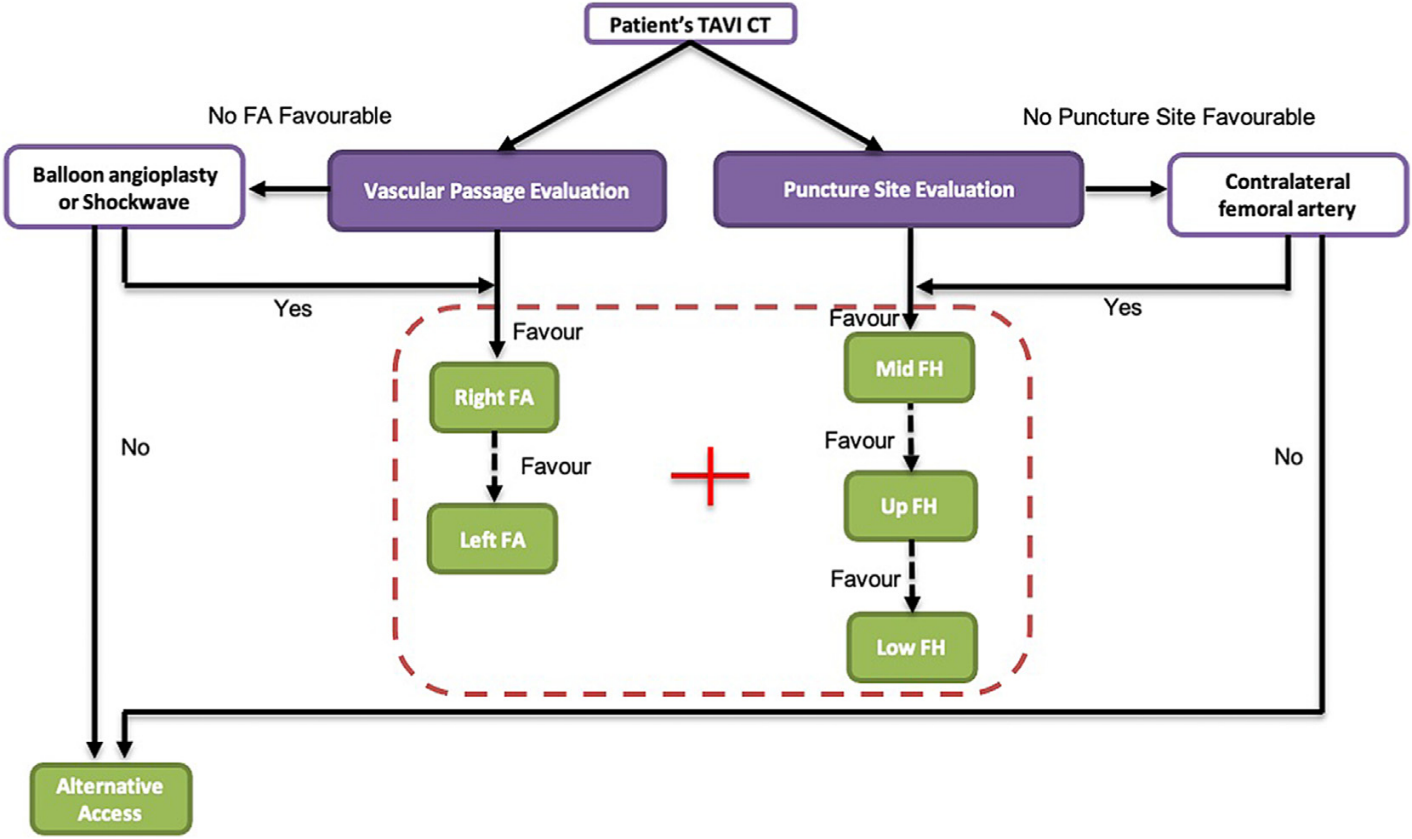
Algorithm on selection of femoral access and puncture site. Abbreviations: CT, computed tomography; FA, femoral access; FH, femoral head; TAVI, transcatheter aortic valve intervention.

For each patient, “passage score,” “puncture score,” and “passage-puncture score” (passage score + puncture score) were calculated, starting from the right femoral artery at the level of the mid-femoral head. If the preferred access vessel or puncture site was associated with a high score or access was deemed not feasible, vessel evaluation was performed in a stepwise manner to the left side for femoral passage and from mid, to lower, to upper femoral head with regard to puncture height (“passage-puncture score,” Figure 2).

### Femoral Artery Access

All patients underwent TAVI using the imaging co-registration technique with pre-TAVI CT and fluoroscopy to identify the predefined puncture site without the use of ultrasound or intraprocedural contrast angiography. The co-registration technique involves^1^: defining the optimized puncture site in the upper, mid, or lower part of the femoral head according to the pre-TAVI CT^2^; palpating the pulse of the femoral artery to define the long axis of the puncture site^3^; placing the needle on the skin at the anticipated puncture height^4^; comparing the needle with the femoral head location under fluoroscopy (in posterior-anterior projection); and adjusting the puncture location to achieve vessel access at the predefined puncture site (Figure 1).

### Suture-Based Vascular Closure

**Figure 3:**
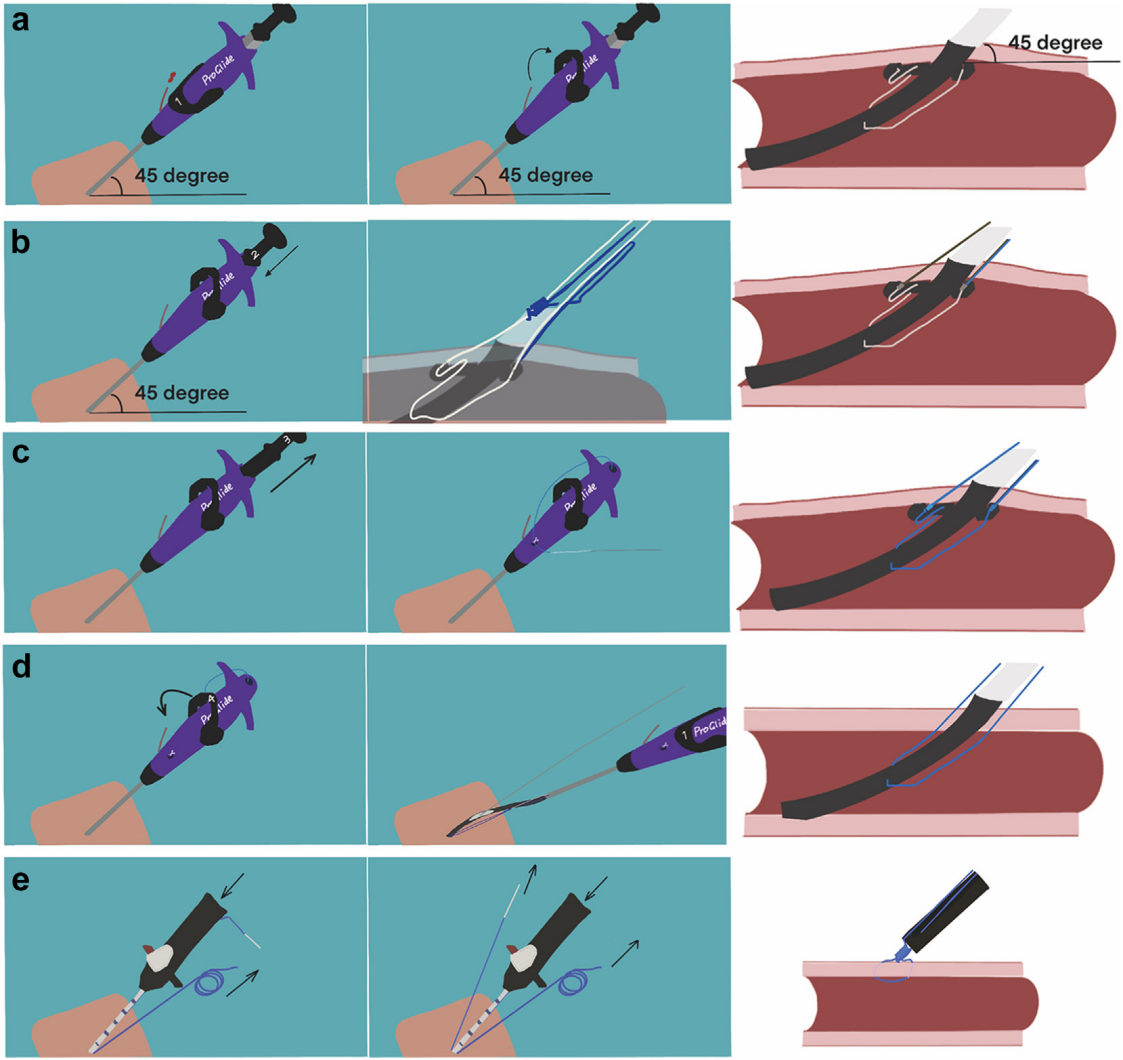
Single ProGlide technique. Illustration of mechanism and technique of ProGlide. (a) Opening of the foot, (b) needle puncture, (c) circling the suture, (d) closing the foot, and (e) locking the knot.

The Perclose ProGlide is a suture-based device implanted using a preclosure technique. After a 45-degree puncture channel was accessed, the ProGlide device was introduced over a J-wire. Then, the lever was pushed back to open the foot and stabilize the device at 45 degrees, and the device was retracted to fully touch the anterior artery wall. The plunger was pushed to deploy the needles to puncture the arterial wall and achieve the needle-suture circular connection. The plunger was pulled back to thread the suture knot. Then, the device was advanced, and the lever was put in the original position to close the foot before backing out of the device. Having removed the sheath after TAVI, the knot was advanced with the suture trimmer and the guidewire removed. Then the suture knot was locked by pulling on the white-tipped suture, and the sutures were cut (Figure 3). If critical bleeding persisted after advancing the suture trimmer, the guidewire was retained to facilitate the insertion of an appropriate sheath to seal the arterial hole. Simultaneously, a crossover technique from the contralateral access and a bailout strategy, such as employing a covered stent, could be considered.

### Outcomes

The primary endpoint was the rate of vascular complications at 30 days, including minor and major vascular complications according to the definitions of the Third Valve Academic Research Consortium.^7^ Secondary endpoints included mortality, need for an additional VCD, need for red blood cell transfusion, stroke, myocardial infarction, renal dysfunction, bleeding, and length of hospital stay.

### Statistical Analysis

Continuous variables are presented as mean ± standard deviation or as median (interquartile range). Categorical variables are presented as counts and percentages. Passage-puncture scores were compared using either a Mann-Whitney *U* test (for unpaired continuous variables) or Wilcoxon matched-pairs signed rank test (for paired continuous variables). Outcomes were compared using either the Mann-Whitney *U* test (for continuous variables) or Pearson chi-square/Fisher’s exact test (for categorical variables). All testing was two-sided, and a two-sided *p* value of <0.05 was considered statistically significant. All statistical analyses were performed using IBM-SPSS version 27 (IBM Corp, NY, USA).

## Results

### Patients and Procedures

**Figure 4:**
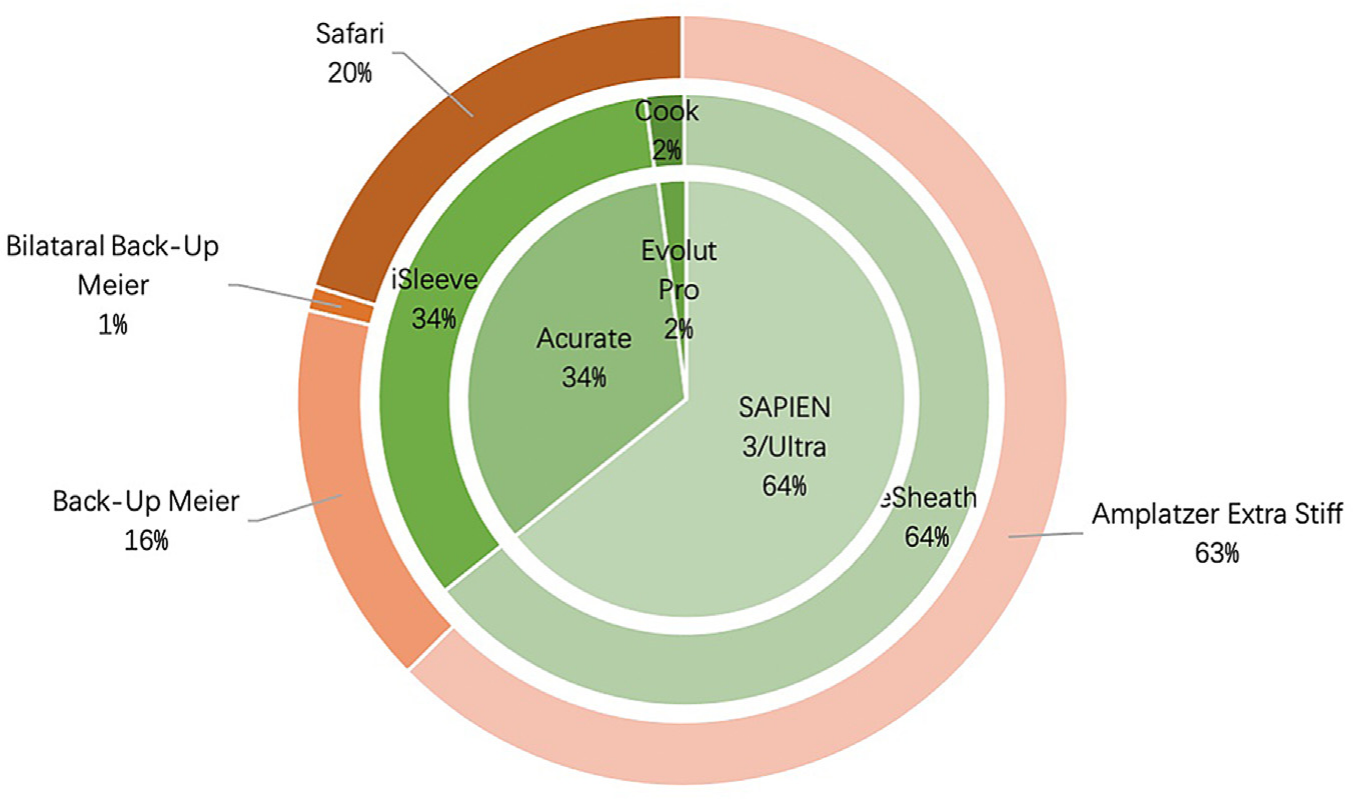
Procedural characteristics. Chart showing the distribution of THV type, sheath, and stiff wire for sheath introduction. Abbreviation: THV, transcatheter heart valve.

Out of 98 patients, 55 (56%) were male, median age was 81 (interquartile range: 75 to 86) years, median European System for Cardiac Operative Evaluation II (EuroSCORE II) was 2.4% (1.6-3.5), median Society of Thoracic Surgeons score was 1.9% (1.3-3.1), and median body mass index was 26 kg/m^2^ (interquartile range: 23 to 30) (Table 1). Baseline and procedural characteristics are summarized in Table 1 and Figure 4.

**Table 1:** Baseline and procedural characteristics

Baseline characteristics	N = 98
Sex, male	55 (56)
Age, y	81 (75-86)
Body mass index, kg/m^2^	26 (23-30)
Hypertension	96 (98)
Dyslipidemia	90 (92)
Type 2 diabetes	32 (33)
Dialysis	2 (2)
Chronic obstructive pulmonary disease	12 (12)
Atrial fibrillation	40 (41)
Peripheral artery disease	21 (21)
Coronary artery disease	68 (69)
Prior PCI	31 (32)
Prior myocardial infarction	17 (17)
Prior stroke/TIA	7 (7)
Prior pacemaker	10 (10)
Prior cardiac surgery	7 (7)
European System for Cardiac Operative Evaluation II, %	2.4 (1.6-3.5)
Society of Thoracic Surgeons score, %	1.9 (1.3-3.1)

Procedural characteristics	N = 98
Minimal femoral artery diameter at puncture site, mm	7.4 ± 1.7
Sheath type	
eSheath	63 (64)
iSleeve	33 (34)
Cook	2 (2)
Sheath size, F	14 (14-16)
Wire	
Amplatzer extra stiff	61 (62)
Back-up Meier	16 (16)
Bilateral Back-up Meier	1 (1)
Safari	20 (20)
THV	
Edwards SAPIEN 3/3 Ultra	63 (64)
Acurate Neo 2	33 (34)
Evolut PRO	2 (2)
Antithrombotic management pre-TAVI	
None	17 (17)
Single antiplatelet therapy	40 (41)
Dual antiplatelet therapy	5 (5)
OAC alone	36 (37)
OAC + antiplatelet therapy	1 (1)

*Notes.* Values are median (interquartile range), mean ± SD, or n (%).

Abbreviations: OAC, oral anticoagulant agent; PCI, percutaneous coronary intervention; TAVI, transcatheter aortic valve intervention; THV, transcatheter heart valve; TIA, transient ischemic attack.

**Table 2:** Outcomes

Discharge outcomes	N = 98
Vascular complications	7 (7)
Access site-related	6 (6)
Major	0 (0)
Minor	6 (6)
Death	1 (1)
Additional vascular closure device used	1 (1)
Bleeding	7 (7)
Need for RBC transfusion	3 (3)
Length of hospital stay, d	8 (5-10)
Stroke	5 (5)
Periprocedural myocardial infarction	1 (1)
Renal dysfunction	5 (5)

*Notes.* Values are median (interquartile range) or n (%).

Abbreviation: RBC, red blood cell.

### Passage-Puncture Score

**Figure 5:**
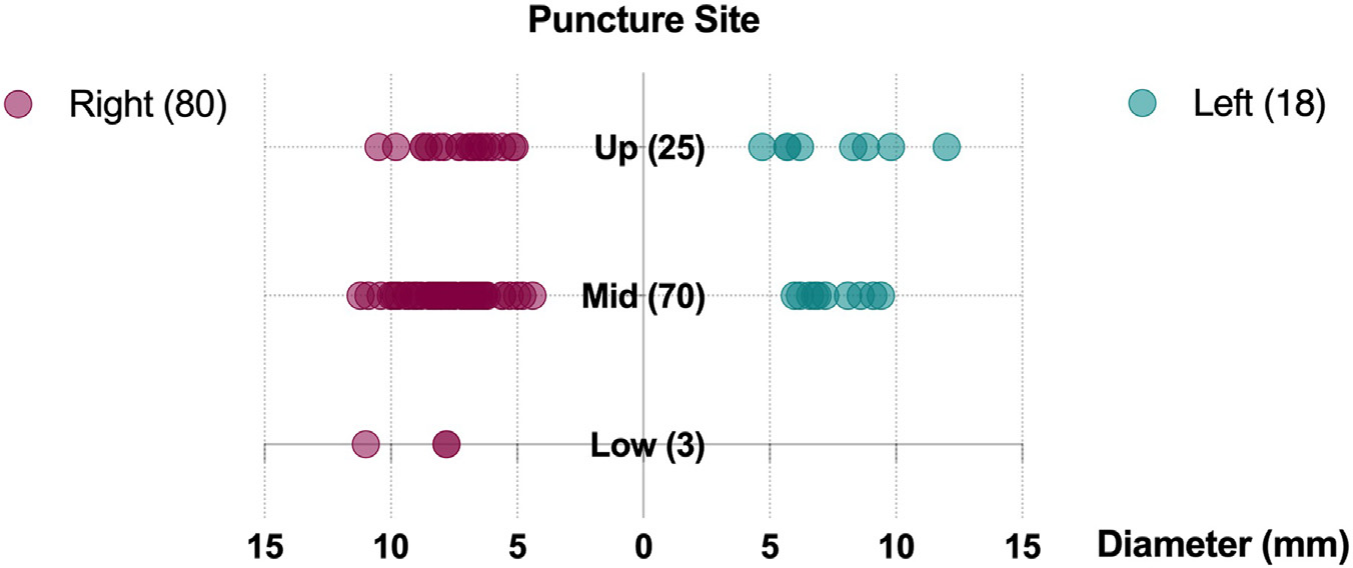
Access side and puncture site selection and its diameter distribution.

**Figure 6:**
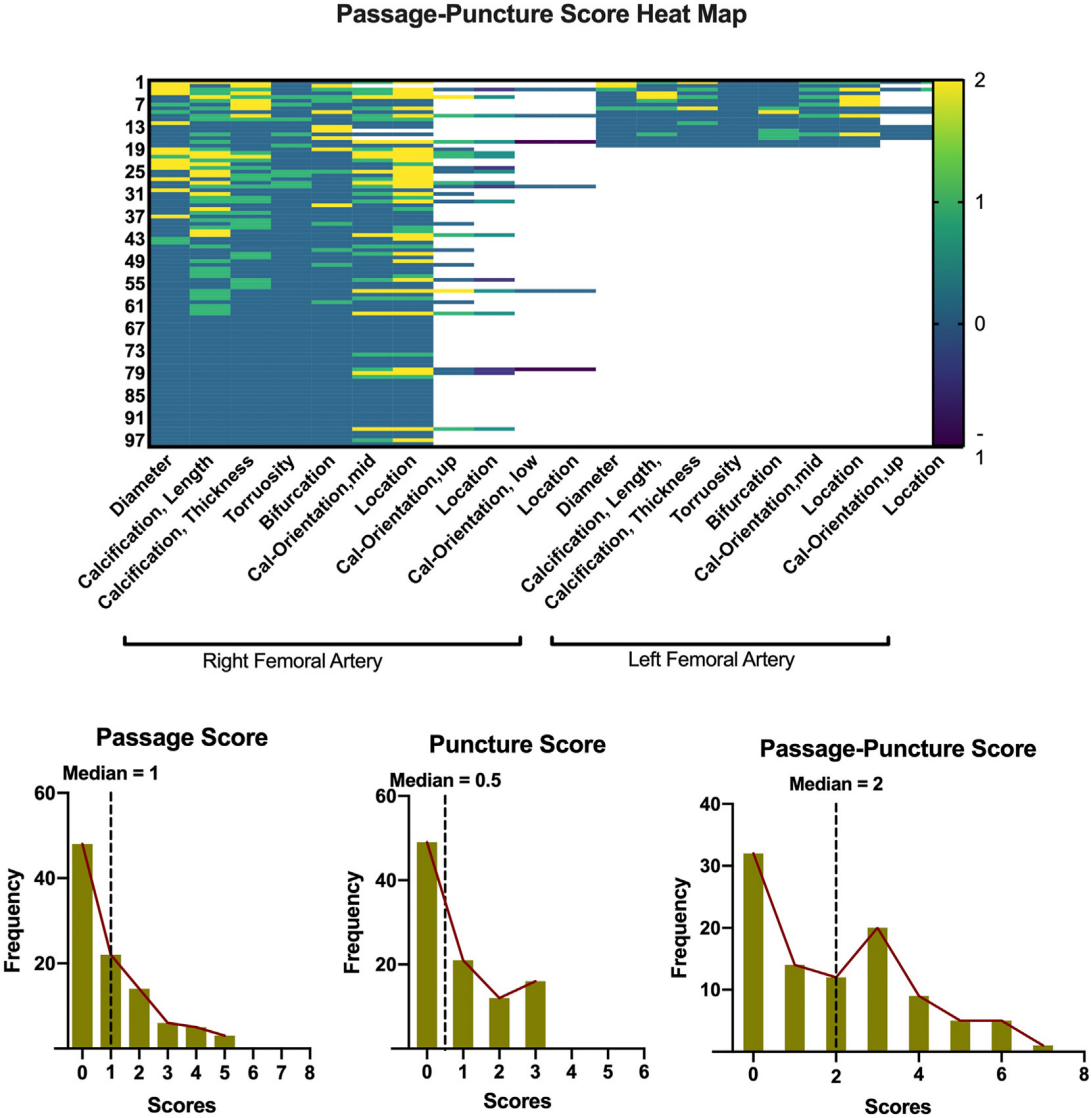
Passage-puncture score distribution. Heat map of each patient’s passage-puncture scores (upper panel) and distribution of passage score, puncture score, and passage-puncture score (lower panel).

Following the passage-puncture algorithm, 80 patients (82%) underwent right femoral artery access, and 18 patients (18%) underwent left femoral artery access. Upper, mid, and low puncture heights were selected in 28 patients (29%), 68 patients (69%), and 2 patients (2%), respectively. The diameters of puncture site are illustrated in Figure 5. The median passage score was 1 (interquartile range 0-2) on the treated femoral side, the median puncture score was 0.5 (interquartile range 0-2) at the puncture site, and the median passage-puncture score was 2 (interquartile range 0-3, Figure 6).

**Figure 7:**
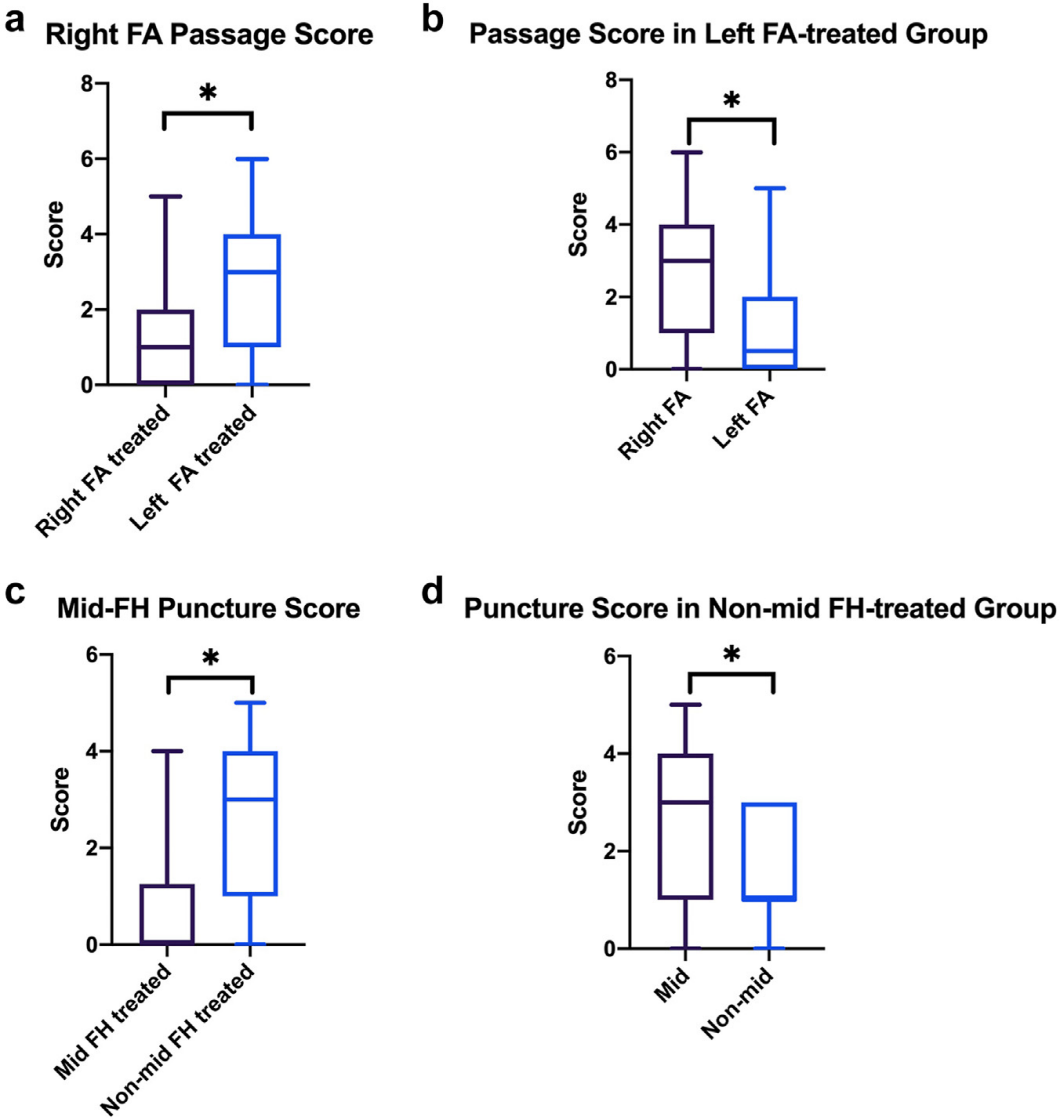
Passage-puncture score in different treatment groups. ∗Denotes statistically significant. (a) Passage score of right FA in patients undergoing right vs. left femoral artery access. (b) Passage score of the right and left femoro-iliacal arteries in patients undergoing left femoral artery access. (c) Puncture score of the mid-femoral artery puncture site in patients undergoing mid-femoral and nonmid-femoral artery punctures. (d) Puncture score of the mid- and nonmid-femoral artery segments in patients undergoing nonmid-femoral artery puncture Abbreviations: FA, femoral access; FH, femoral head.

The passage score of the right femoral/iliac artery was significantly higher in patients treated by left as compared to those treated by right femoral access (FA) (3.0 vs. 1.0; *p* <0.001), whereas the Wilcoxon signed-rank test revealed a significant decreased passage score from the right FA to the left FA in the left FA-treated group (*p* = 0.001, median difference = -1.0), with a large effect size (r = 0.71) (Figures 7a and b).

The puncture score of the mid-femoral artery segment was significantly higher in patients who underwent nonmid-femoral as compared to those who underwent mid-femoral artery puncture (3 vs. 0, *p* < 0.001), whereas nonmid-FA puncture group revealed a significant decreased puncture score from mid-FA to treated puncture site (*p* < 0.001, median difference = -1), with a large effect size (r = 0.67) (Figures 7c and d).

### Outcomes

Access-site-related minor vascular complications occurred in 6 of 98 patients (6%, Table 1 and Supplementary Table) in discharge. Vascular complications comprised pseudoaneurysms (50%), hematoma (33%), and arterial narrowing/occlusion (17%). Out of these patients, 5 patients had >1 point in the passage-puncture score, and 2 patients reached 6 points. Implantation of a covered stent was performed in 1 patient with a passage-puncture score of 6. Red blood cell transfusion was required in 1 patient with passage-puncture score of 3 (Table 1). Secondary outcomes are summarized in Table 1.

**Figure 8:**
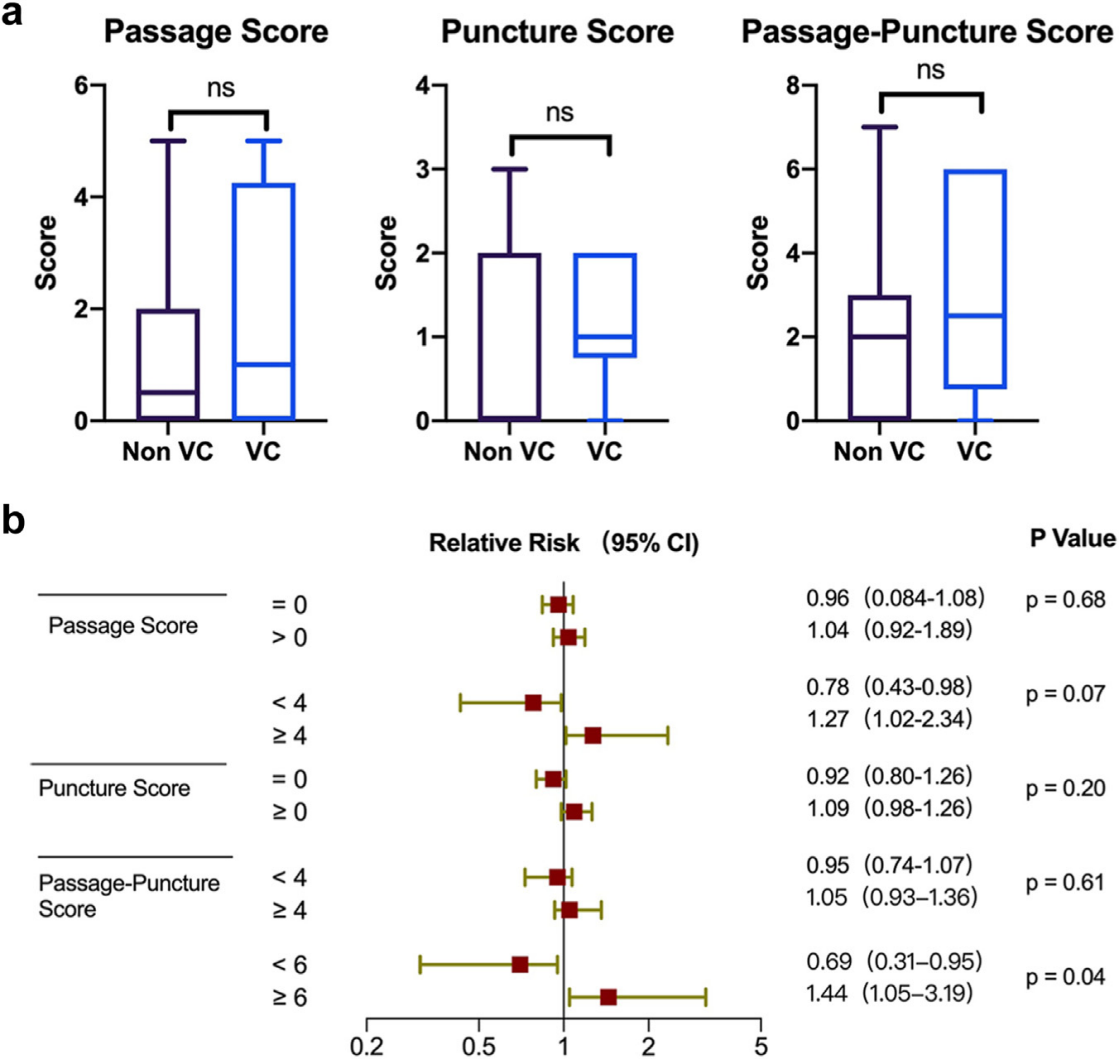
Analysis for access-site-related vascular complications in discharge. (a) Passage score, puncture score, and passage-puncture score were not significantly different between the vascular complications group and the nonvascular complications group. (b) Subgroup analysis for access site-related vascular complications was performed. Relative risk and 95% CI are depicted for the different subgroups Abbreviation: ns, not significant; ROC, receiver-operating characteristic; VC, vascular complications.

Upon systematic evaluation and strategic selection of femoral artery access side and puncture site, both groups—irrespective of overall vascular conditions—achieved comparable passage (scores: 1.0 vs. 0.5, *p* = 0.37) and puncture scores (scores: 1.0 vs. 0.0, *p* = 0.31), demonstrating no statistically significant differences. The passage-puncture score was comparable among groups (2.5 vs. 2.0, *p* = 0.30, Figure 8a). Vascular complications were significantly increased in patients with a passage-puncture score ≥6 (relative risk [RR] = 1.44 (1.05-3.19), *p* = 0.04, Figure 8b).

## Discussion

This is the first study to demonstrate a dedicated algorithm for systematic access site assessment in patients undergoing TAVI. The main findings of this study can be summarized as follows^1^: the passage-puncture algorithm allowed for the identification of an anatomically optimal access site for TAVI, and^2^ access-site related vascular complications were low in patients undergoing TAVI with a systematic assessment of the femoro-iliacal arteries.

Vascular complications during TAVI occur at an estimated frequency of 4.4% to 9.4%.^1^ The sheath-to-femoral-artery ratio has been defined as a predictor for vascular complications.^2^^,^^5^ Access complications are also more frequent with female sex, extremes of weight, renal insufficiency, anticoagulation, and the use of glycoprotein IIb/IIIa inhibitors. Several retrospective studies have associated inappropriate puncture locations with an increased risk of complications. Cannulation above the inguinal ligament is associated with retroperitoneal bleeding, whereas insertion below the common femoral artery bifurcation is associated with pseudoaneurysm and arteriovenous fistula formation.^8^ Several studies have proposed screening procedures for access site selection. Blakeslee-Carter et al.^3^ developed an iliac morphology score to evaluate the prevalence of calcifications and the minimum artery diameter. Durand et al.^5^ scored tortuosity and calcifications of the ilio-femoral axis. To the best of our knowledge, no study to date has demonstrated a systematic method to evaluate transfemoral access for TAVI. The proposed passage-puncture score may assist vessel selection for large bore sheath insertion (passage score) and puncture site selection using suture-based VCD (puncture score).

Fluoroscopy-, ultrasound-, or angiography-guided strategies are the three techniques employed to obtain FA during TAVI. Meta-analysis showed that an ultrasound-guided approach was significantly associated with a reduced risk of access site vascular complications (10.2 vs. 14.8%).^9^ A standard mid-femoral artery puncture under fluoroscopy, without taking into account the presence and distribution of calcifications, may be the main reason for the inferiority of fluoroscopic guidance to ultrasound guidance. Therefore, the strategy proposed in this study systematically integrated the information about vessel dimensions, bifurcation location, and the presence and distribution of arterial calcifications obtained from TAVI-CT with the use of the femoral head as anatomical reference. Our protocol demonstrated a notably superior incidence of minor vascular complications at 6% in comparison to the approach guided by ultrasound. Moreover, the utilization of ultrasound guidance could potentially entail a heightened risk of necessitating manual hemostasis due to the potential loss of femoral reference upon puncture.

Closure of large bore access using two suture-based devices remains the most commonly used closure technique in transfemoral TAVI.^10^ The plug-based Manta (Teleflex, Wayne, Pennesylvania) VCD allowed for vessel closure with a single device; however, randomized controlled data showed fewer vascular complications associated with the ProGlide in comparison to the Manta system.^4^ However, device failure and arterial stenosis are the main concerns of the ProGlide system. Based on our experience developing the “parallel suture technique” and more advanced experience with ProGlide along with puncture site selection, the single ProGlide strategy was adopted and subsequently applied in all patients. The key technique points are^1^: appropriate puncture site selection in TAVI-CT; and^2^ integration of the information obtained by CT with fluoroscopic. A puncture at 45° angulation is important, as a puncture at a too step or flat angulation may interfere with ProGlide deployment. While our study population consisted of a relatively small sample size of 98 patients, it remains, to the best of our knowledge, the biggest single-arm cohort to date with the consecutively employed single-ProGlide technique. The development of the passage-puncture score serves the purpose of facilitating a comprehensive evaluation, enabling operators to assess the feasibility of passage and puncture separately while considering critical evaluation factors. In scenarios characterized by intricate cases or areas where the feasibility of FA or puncture segment remains subject to debate, the meticulous calculation of the passage-puncture score has the potential to offer a refined selection strategy, thereby effectively reducing the incidence of vascular complications.

### Study Limitations

The study is limited by its single-arm, single-center design with a relatively small group of patients, and the proposed algorithm needs prospective validation in an independent cohort. Further, only highly experienced operators performed the procedures in this patient cohort, and the results may not be transferred to a more heterogeneous group of operators performing TAVI.

## Conclusions

The passage-puncture score is an effective method to select the optimal access site for transfemoral TAVI. Such a systematic approach to access site selection may further reduce rates of vascular complications in TAVI.

## Ethics Statement

The study was approved by the Ethics Committee (Cantonal Ethics Committee Zurich) and conducted in full conformance with the Declaration of Helsinki. All patients provided written informed consent.

## Funding

The authors have no funding to report.

## Data Availability Statement

The data underlying this article will be shared at reasonable request to the corresponding author.

## Disclosure Statement

M. Chen received grant from Boston Scientific and she is a consultant for Jenscare Scientific. J. Michel is a proctor for Boston Scientific. B. E. Stähli received grants to the institution from Edwards Lifesciences and Boston Scientific. A. M. Kasel is a consultant and proctor for Edwards Lifesciences and received grants to the institution from Edwards Lifesciences.

The other authors had no conflicts to declare.
