# Supplementary material for: A Novel Risk Score Facilitates Femoral Artery Access in Transcatheter Aortic Valve Implantation: Passage-Puncture Score
Source: Struct Heart. 2024 Jun 25;8(5):100331. doi: 10.1016/j.shj.2024.100331 (PMC11403084; doi:10.1016/j.shj.2024.100331)
Supplement: Supplementary Table 1 [file mmc1.docx]

Supplementary Table: Vascular Complications

| **Patient** | **Age** | **Sex** | **Passage Score** | **Puncture Score** | **Passage-Puncture Score** | **THV** | **Sheath** | **Major/Minor vascular complications** | **Categories** | **Management** |
| --- | --- | --- | --- | --- | --- | --- | --- | --- | --- | --- |
| 1# | 84 | F | 1 | 2 | 3 | 23# Acurate | 14 Fr iSleeve | Minor | Pseudoaneurysm | Thrombin injection |
| 2# | 82 | F | 4 | 2 | 6 | 25# Acurate | 14 Fr iSleeve | Minor | Hematoma | Compression |
| 3# | 79 | M | 0 | 0 | 0 | 29# Sapien | 16 Fr eSheath | Minor | Pseudoaneurysm | Compression |
| 4# | 89 | F | 5 | 1 | 6 | 23# Acurate | 14 Fr iSleeve | Minor | Stenosis | Stent |
| 5# | 90 | F | 1 | 1 | 2 | 23# Sapien | 14 Fr eSheath | Minor | Hematoma | None |
| 6# | 90 | M | 0 | 1 | 1 | 26# Sapien | 14 Fr eSheath | Minor | Pseudoaneurysm | Compression |
